# [Retracted] Clinical significance of miR‑1298 in cervical cancer and its biological function *in vitro*

**DOI:** 10.3892/ol.2024.14283

**Published:** 2024-02-12

**Authors:** Haitao Zhang, Ruihong Zhang, Guiling Zhang, Wenjuan Liu, Zhaoyuan Ma, Caiyun Yue, Min Yang

Oncol Lett 21: 401, 2021; DOI: 10.3892/ol.2021.12662

Following the publication of this paper, it was drawn to the Editor's attention by a concerned reader that certain of the Transwell cell migration and invasion data shown in Fig. 3C and D on p. 5 were strikingly similar to data that had appeared in different form in different articles written by different authors that either had already been published, or were under consideration for publication at around the same time.

Owing to the fact that some of the data in the above article had already been published prior to its submission to *Oncology Letters*, the Editor has decided that this paper should be retracted from the Journal. The authors were asked for an explanation to account for these concerns, but the Editorial Office did not receive a reply. The Editor apologizes to the readership for any inconvenience caused.

